# Myocardial perfusion and viability after percutaneous recanalization of coronary chronic total occlusions: a cardiovascular magnetic resonance study

**DOI:** 10.1186/1532-429X-11-S1-P152

**Published:** 2009-01-28

**Authors:** Chiara Bucciarelli-Ducci, Didier Locca, Giuseppe Ferrante, Joanna Petryka, Agata Grasso, Rory O'Hanlon, Francesca Del Furia, Christine Wright, Karen Symmonds, Peter Gatehouse, Sanjay Prasa, Carlo Di Mario, Dudley Pennell

**Affiliations:** 1 CMR Unit, Royal Brompton Hospital, London, UK; 2 Interventional Cardiology, Royal Brompton Hospital, London, UK

**Keywords:** Myocardial Perfusion, Cardiovascular Magnetic Resonance, Myocardial Perfusion Imaging, Chronic Total Occlusion, Myocardial Perfusion Reserve

## Background

Coronary angiography can assess the anatomy of collaterals but is of limited value for evaluating their functional significance. In clinical practise, the size of collateral connection correlates is assessed by 3 grades: CC0, no continuous connection between donor and recipient artery; CC1, continuous, thread-like connection; and CC2, continuous, side branch-like connection. Its direct validation by myocardial perfusion imaging has not been established so far. The aim of this study was to assess the impact of collateral connection on infarct size and myocardial perfusion reserve by adenosine cardiovascular magnetic resonance (CMR) perfusion imaging in patients with single-vessel chronic total occlusion (CTO).

## Methods

Forty-two consecutive patients with CTO (occlusions older > 3 months) underwent adenosine CMR perfusion prior to percutaneous coronary intervention. Grading of collateral connection (CC0-CC2) was carried out in multiple angiographic projections prior to recanalization. Myocardial perfusion reserve index (MPRI) (cut-off of normality ≥ 2), the amount of infarcted area, and the number of non-viable segments were calculated and corrected for the dimension of area at risk. Data are expressed as mean ± SD or median and range (min-max), as appropriate.

## Results

The angiographic grading demonstrated that 11 patients had no continuous connection (CC0), 14 patients had continuous thread-like connection (CC1) and 17 patients continuous side branch-like connection (CC2). In 75% of the patients MPRI was reduced (<2). MPRI >2 was present in 25% of patients and its prevalence significantly increased from 0% to 23.1% to 43.7% in CC0, CC1 and CC2 respectively, p = 0.03.

**Figure 1:**
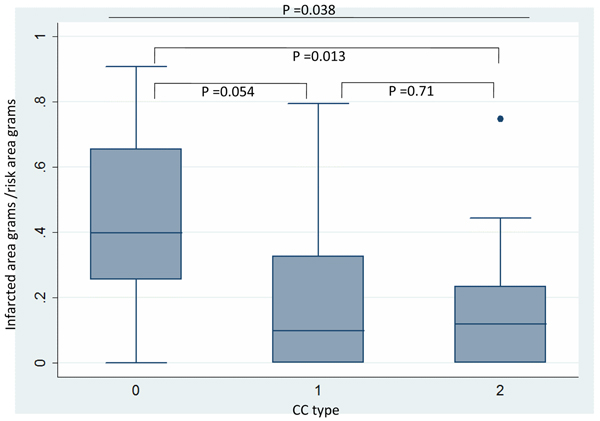
Figure 1

The percentage of infarcted myocardium in the area at risk was significantly different among the three groups: 39% (0–91%) in CC0, 9% (0–79%) in CC1 and 12% (0–75%) in CC2, p = 0.038 (p = 0.0128 for comparison CC0 vs CC2, p = 0.054 for CC0 vs CC1, p = 0.71 for CC1 vs CC2). (See figure 1) Patients with CC0 had a higher percentage of non-viable segments compared with patients with CC1 and CC2 (48% ± 41 vs 22% ± 54, p = 0.04).

## Conclusion

In patients with CTO the presence of CC2 (more developed collaterals) is more frequently associated with smaller infarct size and a lower number of non-viable segments in the risk area. More developed collaterals are also associated with a higher prevalence of normal myocardial perfusion reserve in the area at risk. CMR perfusion is a useful non-invasive method to assess the functional significance of collateral connections.

